# Changes of frontostriatal connectivity in the resting-state functional magnetic resonance imaging of alcohol use disorder patients

**DOI:** 10.1192/j.eurpsy.2026.10812

**Published:** 2026-07-31

**Authors:** N. Fan, X. Song, J. Chen, W. Zou

**Affiliations:** The Affiliated Brain Hospital, Guangzhou Medical University, 36 Mingxin Road, Liwan District, Guangzhou, Guangdong Province, China

## Abstract

**Introduction:**

Alcohol use disorder (AUD) is a prevalent global health issue, affecting individuals across all socioeconomic backgrounds, yet the underlying mechanism remains poorly delineated. Growing evidence revealed dysfunction in frontostriatal functional connectivity (FC) in AUD, particularly involving impaired cortical regulation of alcohol use behaviors.This study aims to investigate changes of frontostriatalconnectivityin AUD and their correlation with cognitive deficits.

**Objectives:**

To investigate alterations in frontostriatal functional connectivity in male patients with alcohol use disorder (AUD) and examine the relationship between these neural changes and cognitive deficits, as well as clinical measures of alcohol dependence and craving.

**Methods:**

Sixty-six male patients with AUD and 43 male healthy controls (HC) were recruited. Alcohol dependence and craving were evaluated using the Alcohol Use Disorder Identification Test(AUDIT), Visual Analog Scale (VAS), Obsessive-Compulsive Drinking Scale-5(OCDS), and Alcohol Urge Questionnaire(AUQ). Cognitive function was assessed using tasks from MATRICS Consensus Cognitive Battery(MCCB). Seed-based FC analysis was performed using bilateral dorsolateral prefrontal cortex (DLPFC), nucleus accumbens (NAc), and dorsal striatum (DS) as seeds regions and correlated with clinical scale scores.

**Results:**

Compared to HC, AUD patients scored lower in speed of processing, working memory and problem-solving (*P*<0.05). AUD patients exhibited reduced FC between bilateral DLPFC and the orbital inferior frontal gyrus, medial orbital gyrus, superior frontal gyrus, angular gyrus,middle temporal gyrus, inferior temporal gyrus, and cerebellum. AUD patients showed decreased connectivity between bilateral DS and the middle frontal gyrus, inferior frontal gyrus, supramarginal gyrus, and cerebellum. Left NAc showed reduced connectivity with the middle occipital gyrus, while right NAc exhibited decreased connectivity with the middle frontal gyrus, anterior cingulate gyrus, angular gyrus, and precuneus. Correlational analysis revealed that reduced FC of the DLPFC was positively correlated with craving scores and negatively correlated with cognitive performance. FC between left DS and right supramarginal gyrus was negatively correlated with VAS scores(r=-0.268, P=0.030), and FC between right DS and right supramarginal gyrus was positively correlated with verbal learning scores(r=0.275, *P*=0.040).

**Conclusions:**

Patients with AUD exhibit widespread cognitive impairments and may correlated with reduced connectivity of DLPFC and cortical-striatal hypoconnectivity.

**Disclosure of Interest:**

None Declared

